# Solo songs, duets and territory defence across seasons in female Galápagos yellow warblers, *Setophaga petechia aureola*

**DOI:** 10.1016/j.anbehav.2026.123483

**Published:** 2026-04

**Authors:** Alper Yelimlieş, Katherine Albán Morales, Çağlar Akçay, Sonia Kleindorfer

**Affiliations:** a Konrad Lorenz Research Center for Behavior and Cognition, Grünau im Almtal, Austria; b Department of Behavioral and Cognitive Biology, University of Vienna, Vienna, Austria; c Ecology, Evolution and Environment Research Centre, Anglia Ruskin University, Cambridge, U.K.; d School of Life Sciences, Anglia Ruskin University, Cambridge, U.K.

**Keywords:** aggression, birdsong function, female song, Galápagos yellow warbler, *Setophaga petechia aureola*

## Abstract

Although the function of birdsong has been well studied in male songbirds, the function of female song is less well understood. This is partly due to a historical view of females occupying a passive role compared with males, which led to the neglect of female song. We examined the female song in Galápagos yellow warblers, *Setophaga petechia aureola*, and show for the first time that it is widespread in the form of solo songs as well as duets. We interrogated the seasonal patterns and functions of female song by carrying out simulated territory intrusions using playbacks of male, female or duet songs during breeding and nonbreeding seasons and conducted a territory retention survey for over a year. We measured the association between aggressive response and singing behaviour, sex-specific patterns of response and territory retention across years. Females sang mostly during the nonbreeding season and predominantly in male-led duets. Although females were strongly aggressive towards female song playback, they gave the weakest singing response to it. There was no association between female aggressive behaviour and song output in response to a simulated intruder. Moreover, the probability of territory retention across years was not explained by song output or aggression in response to intruders, though evidence for this was weak due to the small sample size. We suggest that female song in this year-round resident island system does not function for signalling aggression in territory defence or intrasexual competition but may have other functions.

The study of sexual selection has largely focused on male ornaments while neglecting females. This biased focus has been compounded historically by a longstanding view of females occupying a passive role compared with males (Ah-King, 2022b; Dougherty, 2021; Hrdy, 1986), which consequently has impeded the progress in our understanding of the evolutionary history and significance of female ornaments. More recently, with the inclusion of feminist perspectives into research, this view is being replaced by one in which females are active participants in inter- and intrasexual selection (Ah-King, 2022a; Tang-Martínez, 2020).

Research on birdsong is a good example of this historical trajectory. Although females of some species were long known to sing, female song was viewed as an exceptional phenomenon, mostly occurring in the tropics or as a byproduct of abnormal testosterone levels (Catchpole & Slater, 2008). Increased attempts to address this bias in the last decades revealed that singing females are not exceptions; rather, female song is estimated to be an ancestral trait in songbirds (Odom et al., 2014) and present in 59% of the songbird species (Odom et al., 2025). Despite the significant progress, the current state of knowledge is nonetheless incomplete, as we lack sex-specific singing information on 73% of the songbird species (Odom & Benedict, 2018).

Increased research on female song has revealed both convergent and divergent patterns of singing and song structure between sexes (Evans & Kleindorfer, 2016; Liu et al., 2024; Magoolagan et al., 2019; Moyer et al., 2025; Patchett et al., 2021; Sierro et al., 2022). The divergent structural patterns suggest functions of singing may be sex specific in at least some species (Morton, 1977); therefore, a complete understanding of birdsong function requires separate investigation of both males and females (Barros et al., 2024).

The ecological significance of song is primarily explained by its dual function: mate attraction and territory defence (Catchpole & Slater, 2008). To date, these functions have mostly been tested in males, who sing to attract mates and defend their resources against same-sex intruders (Eriksson & Wallin, 1986; Krebs et al., 1978). Studies on female song generally supported the same dual function (Beletsky, 1983; Krieg & Getty, 2016; Langmore et al., 1996). Additionally, females of some species use song in within-pair (Beletsky, 1983; Halkin, 1997; Rose et al., 2019) or parent—offspring communication (Ritchison, 1983). Among these functions, territory defence in particular is the most common function for both solo female songs and duets (Barros et al., 2024; Hall, 2004). In line with this, territoriality also predicts the presence of female singing in multispecies analyses (Mikula et al., 2020; Odom et al., 2025). Moreover, both studies found that among the species with female song, duetting was more common in year-round territorial species than seasonally territorial species.

Studying the recently split *petechia*—*aestiva* complex offers valuable insight into how differences in life history may shape female singing behaviour (AviList Core Team, 2025). Unlike the well-studied migratory North American yellow warbler, *Setophaga aestiva*, the Galápagos yellow warbler, *Setophaga petechia aureola*, is sedentary and territorial year-round (Snow, 1966). Hobson and Sealy (1990) previously reported that female song is rare and occurs only as solo song in the migratory North American species. Their observations suggested that territorial resource defence against conspecific females during the early breeding season could be the main function of female song. In contrast, our observations show that females of the nonmigratory Galápagos yellow warblers commonly produce both solo songs and duets with their male partners. To our knowledge, after the recent taxonomic split, this study provides the first documentation of female song in *S. petechia*.

Phylogenetic estimates indicate that the *aureola* subspecies of *S. petechia* colonized the Galápagos Archipelago approximately 300 000 years ago, whereas the divergence between *S. petechia* and *S. aestiva* likely occurred around 1.4 million years ago (Chaves et al., 2012). In Galapagos, males and females long-term pair bonds, and their territories are stable across years (see Results). Three cases of polygyny were documented in our population, extending into the nonbreeding season. Galápagos yellow warblers inhabit both dense *Scalesia* forests of highlands and shrub habitats in the lowlands of the archipelago. Breeding typically begins after the onset of the rains in January and continues throughout the rainy season, which can be variable on the Galápagos but typically extends until April. Snow (1966) observed breeding activity from December to April on Santa Cruz Island. Pairs can raise one or more broods per season, with incubation and feeding each lasting about 10—14 days, followed by postfledging parental care. Females build the nest and incubate the eggs, while both sexes feed the chicks. In the field, males and females can be readily distinguished by both song and plumage. Males have a rufous crown and rufous chest streaking over their bright yellow and olivaceous plumage, whereas females lack the crown and show little to no streaking (see Fig. 1 for illustrations). Female songs typically contain more repetitive elements and sound harsher than male songs to the human ear. In addition, males and females frequently combine their songs into simultaneous duets (see Fig. 1 for spectrogram examples).

Here, we tested two nonmutually exclusive hypotheses for female solo songs and duets in the Galápagos yellow warbler, namely, intrasexual competition and territory defence. To this aim, we carried out simulated territory intrusion experiments with focal pairs in breeding and nonbreeding seasons by broadcasting solo male, solo female and duet songs. We also conducted separate surveys to compare the probability of territory retention depending on the song rate from June 2023 to March 2025. If female song functions primarily in intrasexual competition, we expect resident females to respond with more aggression and singing to intruder females than males. If female song functions in signalling aggression during territorial encounters (that is, defence of physical resources against both males and females or an attempt to usurp resources), we predict that: (1) playback of female song will elicit equally aggressive responses from both the male and the female, and responses will be comparable to playback of male song; (2) resident female aggressive responses in response to playback of either male or female song will be associated with her song output. Additionally, we predict that (3) pairs will respond more strongly to the duetting intruders, as an intruding pair is assumed to be a stronger threat to territory ownership than a single bird of either sex. Finally, if female song functions in territory defence, we predict that females that are singing more and are more aggressive will be more likely to retain their territory across years. We also report on the occurrence of female singing during the breeding and nonbreeding seasons.

## Methods

### Study Site

We studied the Galápagos yellow warblers on Floreana Island in the Galápagos archipelago between June and July 2023 (nonbreeding season) and between January and March 2024 (breeding season) on multiple sites including Cerro Pajas (−1.295927, −90.456195; highlands), Asilo de La Paz (−1.313046, −90.454748; highlands) and Puerto Velazco Ibarra (the only settlement on the island, −1.275715, −90.486968; low-lands), which have been a part of long-term research on songbirds by our group since 2004 (Kleindorfer et al., 2021; see Fig. S1 for the playback locations).

### Playback Experiment

#### Stimulus preparation

To make the playback stimuli, we recorded male and female songs in June 2023 at a 48 kHz sampling rate and 24 bit resolution using a Sennheiser MKE 600 directional microphone attached to a Zoom H5 recorder. From our recordings, we selected 10 high-quality male and female songs from different individuals and further processed them in Audacity version 3.3.3. Eleven of those 20 individuals were colour banded; for the rest, we recorded from territories that were at least 200 m apart to avoid recording the same individual. First, we high-pass filtered the recordings above 1500 Hz and then normalized the peak amplitude. Then, we created solo male and solo female stimulus files by repeating the same song at a rate of 6 songs per minute. This corresponds to how males sing during agonistic interactions (Beebee, 2004), and we chose to keep it the same for females to standardize the repetition rate. For the duet stimuli, we combined the songs of one male and one female, whereby the female song followed the male song 0.5 s after it had ended. This response delay is within the natural limit of the species’ duets, and we chose to keep it constant across iterations for the sake of simplicity.

#### Experimental procedure

We determined active territories by observing singing posts and the territorial behaviour of the pair (described in Hohl et al., 2025). Upon arrival of a pair in the territory, we placed a Sony SRS-XB100 Bluetooth speaker in a central location at 1.5 m above the ground. During the breeding season, we placed the speaker 10 m away from the nest and within the territory to minimize the disturbance to the breeding activity and to make our response variables relative to the speaker’s position more valid. Then, we marked 1 and 3 m distances from the speaker with flagging tape in two directions to assist with our distance estimates. To start the trials, we waited until both sexes were within 20 m of the speaker, confirmed either by sight or hearing. Then we started the playback period, which consisted of 1 min of song playback followed by 1 min of silence and another minute of song for a total of 3 min. The broadcast amplitude of the stimuli for both sexes was set to ~80 dB measured using a sound level meter (Svan 971, Svantek U.K., A-weighting, fast setting) at 1 m. Although we did not quantify the source level of the song in the field, this amplitude was previously used for playback experiments on North American yellow warblers (Beebee, 2004). During the playback period, we narrated the behaviour of the pair and recorded their vocalizations using a Marantz PMD660 recorder with a Sennheiser ME66/K6 shotgun microphone or a Zoom F3 recorder with a Sennheiser MKE 600 shotgun microphone. After the playback period was over, we recorded the vocalizations of the focal pair for another 5 min. All observations were done by two experienced field researchers (either A.Y., C.A. or K.A.M.); one narrated the behaviour of the male and the other narrated the behaviour of the female, to ensure all behaviour was recorded with as little error as possible.

We tested 17 pairs in the nonbreeding season (June—July 2023) and 20 pairs in the breeding season (January—March 2024) from 29 unique territories. Nineteen of 34 individuals in the nonbreeding season and 14 of 40 individuals in the breeding season were colour banded (see Table S1 for a detailed break-down). Eight territories were tested in both seasons, two of which contained unbanded pairs. Although we could not assign individual identities for these pairs, we assumed that the same individuals occupied the same territories across seasons. This assumption did not affect any of our results qualitatively. For each trial, we used songs from a non-neighbouring individual and avoided testing neighbouring pairs on the same day. Each pair was tested three times, with solo male song, solo female song and duet song stimuli, on the same day. The duet stimulus for each pair contained songs of the same male and female from solo playbacks. The order of trials was randomly chosen (see Table S2 for the distribution of treatment orders), and consecutive trials of the same pair were separated by at least 45 min. All playback trials were conducted during periods of high activity, between 0600 and 1130 hours.

#### Quantifying aggressive and song response

We used Audacity version 3.3.3 to annotate our recordings of trials. From the annotations, we extracted the number of flights, the closest approach to the speaker, time spent within 5 m of the speaker, and latency to respond to playback for the 3 min playback period as proxies for physical aggression. All these variables are commonly used in simulated intrusion experiments of songbirds as indicators of territorial aggression (Searcy et al., 2006) and to predict attack on a taxidermic model (Akçay et al., 2013). We also annotated the number of songs for each bird and duets for each pair for the 3 min playback period and the following 5 min post playback. We defined duets as consecutive songs from both sexes in which the response song starts after the initial song and at the latest two seconds after the initial song ends. If the initiator of the duet sang again, overlapping the responder or within 2 s after the end of the response song, we counted these consecutive songs as one duet. For our analyses of song, we first categorized individuals as singers and nonsingers based on whether they sang any solo or duet songs in response to any of the treatments in each season.

### Territorial Retention Surveys

To quantify the relationship between song rate, aggression, and territory retention, we conducted territory surveys. In 2024 and 2025, we visited each territory with banded birds and coded the female as absent or present. More specifically, 18 banded females were observed from 2023 to 2025, and 8 females were observed from 2024 to 2025. In the cases in which the bird was absent, we confirmed that another unbanded individual of the same sex was defending the territory by doing playbacks in various locations around the territory. For our analyses on aggression and song rate, we used nine of the banded females tested in our playback experiment in the nonbreeding season, which were also included in our surveys.

### Data Analysis

All statistical analyses were conducted in R version 4.3.3 (R Core Team, 2024). As the aggression variables (number of flights, closest approach distance, latency to respond and time spent within 5 m of the speaker) were correlated, we performed a principal component analysis (PCA) to reduce dimensions using the ‘prcomp’ package with centred and scaled variables. We used the ‘PCAtest’ package with 1000 permutations and bootstrap replication to evaluate the significance of the PCA and the resulting PCs (Camargo, 2022). PC1 was significant, accounted for 67.5% (95% CI: 64.2—71.1) of the total variation in the data and had an eigen-value of 2.70. All variables had significant loadings on PC1 (factor loadings shown in Supplementary Table S3). We took the inverse scores as our aggression variable so that the higher values for this variable indicated higher aggression.

We analysed the resulting aggression scores (PC1) using a linear mixed model with Gaussian error distribution using the R package ‘lme4’ (Bates et al., 2015). The model included season (breeding, nonbreeding), sex (male, female), treatment (male, female, duet), all of their two-way interactions, the three-way interaction between them as the fixed effects, and bird ID nested within pair ID as the random effects. In the initial model, we controlled for the effect of treatment order by adding it as a fixed factor, but as it did not reveal a significant effect, we removed it from the final model.

We first wanted to quantify individuals who sang at least one song (solo or duet) in response to any of the treatments in both seasons. To test if the number of singing individuals depends on the season, we performed a χ^2^ test. We performed the test only for the females because all the males were singing in both seasons.

As our test revealed that females were mostly singing in the nonbreeding season (see Results), we restricted our analyses of the number of solo songs and duets produced during the playback and post-playback periods to the nonbreeding season. We first calculated descriptive statistics such as the proportion of solo songs each sex produced and the proportion of duets led by either sex. Then, we modelled each of these two response variables using generalized linear mixed models (GLMMs) with Poisson error distribution and log link function using the package ‘lme4’ (Bates et al., 2015). The number of solo songs model initially included sex, treatment, their interaction, and the treatment order as the fixed effects, and bird ID nested within pair ID as the random effects. As the treatment order effect was not significant, we removed the term from the final model. Because duetting was a pair-level response, the corresponding model included treatment and treatment order as the fixed effects and pair ID as the random effect.

We then tested whether singing is associated with aggression by modelling whether the aggression scores during playback trials predicted songs produced posttrial using a zero-inflated GLMM with Poisson error distribution and log link function using the package ‘glmmTMB’ (Brooks et al., 2017). As females were mainly singing in the nonbreeding season, we excluded the breeding season from the model. For the males, both seasons were used. The model had the songs produced after the trial as the response variable, aggression score (PC1), sex, and their interaction as the fixed factors and bird ID as the random factor. The zero-inflated part of the model included sex as the fixed factor and bird ID as the random factor because we expected zeroes mainly resulting from females.

Finally, we modelled the relationship between song rate and territory retention using the data from our territory surveys. For this, we used a GLMM with a binomial error distribution, which had the territory retention in 2025 (0 = lost, 1 = retained) as the response variable average number of songs across the three playback trials as the predictor variable. We also modelled the probability of territory retention as a function of average aggression score over three trials.

We checked model diagnostics for all the models above using the package ‘DHARMa’ (Hartig, 2024). We report predictor estimates, 95% CIs, as well as χ*2* and *P* values for overall predictors from type 3 ANOVA using the package ‘car’ (Fox & Weisberg, 2019). When needed, we also report Tukey *P* value-adjusted post hoc comparisons using the package ‘emmeans’ (Lenth & Piaskowski, 2025). We used the package ‘ggeffects’ to create the plot for the relationship between aggression and singing (Lüdecke, 2018).

### Ethical Note

This study followed the ASAB/ABS Guidelines (ASAB Ethical Committee/ABS Animal Care Committee, 2026), and complied with all current Austrian laws and regulations and was supported by Animal Experiment Licence Number 66.006/0026-WF/V/3b/ 2014 issued by the Austrian Federal Ministry for Science and Research (EU Standard, equivalent to the university Animal Ethics Board). Permission to conduct this study as well as banding birds was granted by the Galápagos National Park Directorate (permit numbers PC-87-23 and PC-05-25). Subjects were captured with mist nets and banded with a metal numbered band and two or three colour plastic bands as part of the long-term banding programme under these permits. Each bird captured was released within 30 min of capture after banding by an experienced bander. We did not capture the birds again for the experiment. Each trial took 8 min, which included only 2 min of active playback to minimize the stress for the birds. The birds returned to their normal activities within a few minutes.

## Results

### Responses to the Playback Experiment

#### Aggressive behaviour

**Figure 2:**
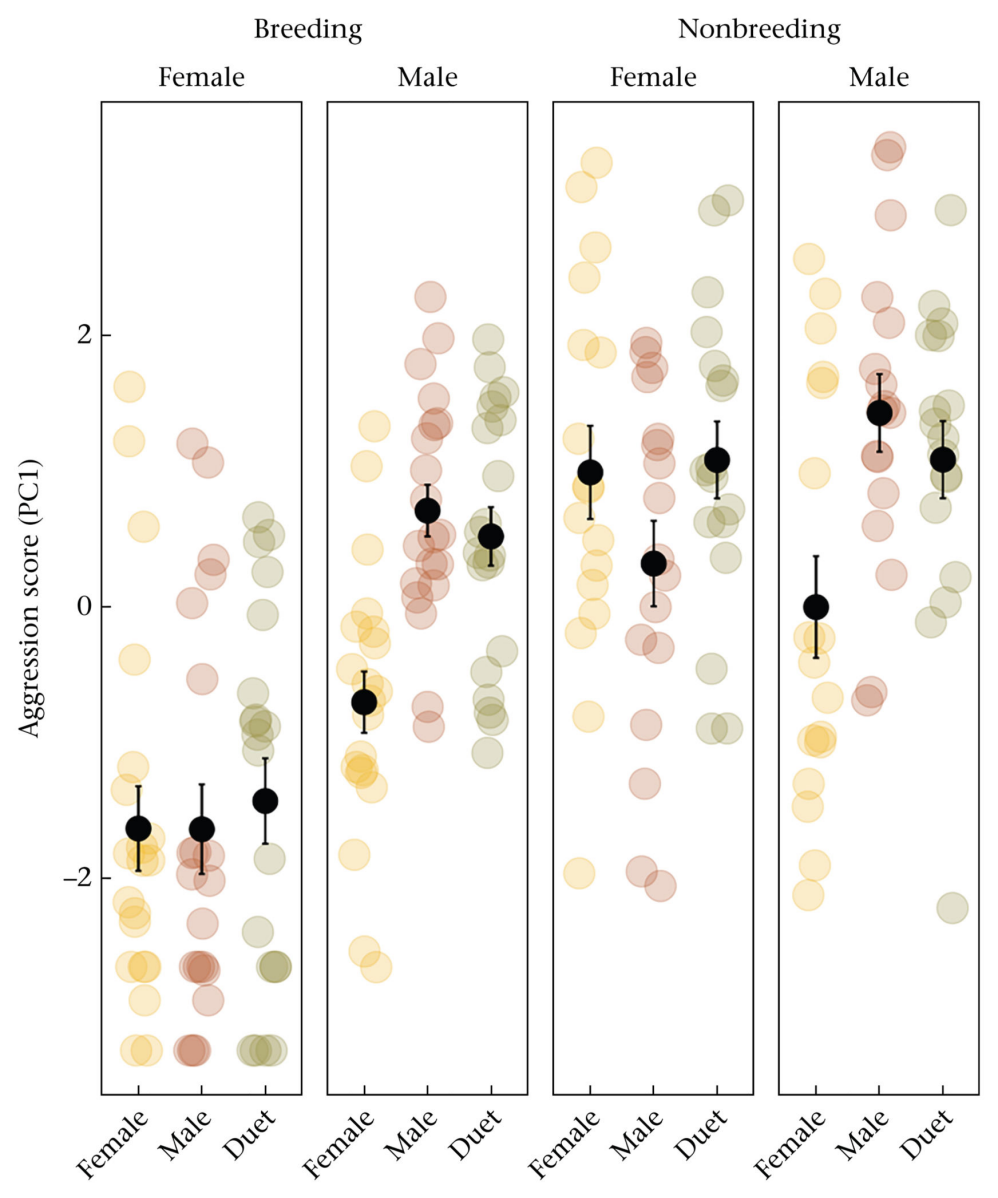
Aggression scores in resident females and males in response to female playback, male playback, and duet playback in the breeding and nonbreeding seasons. Coloured dots show each individual; black dots and whiskers show the group mean and SE.

Aggressive responses to simulated intruders differed between treatments, sexes and seasons. There was a significant interaction effect between sex and season on aggression scores (χ^*2*^ = 9.53, *P* < 0.001), with less aggression in females in the breeding season than in the nonbreeding season, whereas males were equally aggressive in both seasons (Table 1, Fig. 2). There was also a significant interaction effect between sex and treatment (χ*2* = 11.19, *P* = 0.004). Although the female responses to the intruder treatments were higher overall during the nonbreeding season than the breeding season, they did not differ across intruder treatments in either season (all *P* > 0.2, Table S4). By contrast, males responded significantly more to solo male and duet intruders than to solo female intruders in both seasons (breeding: *P* < 0.001, *P* = 0.002, respectively; nonbreeding: *P* = 0.001, *P* = 0.02). Males responded at similar levels to solo male and duet treatments (breeding: *P* = 0.99; nonbreeding: *P* = 0.92).

#### Singing behaviour

**Figure 3:**
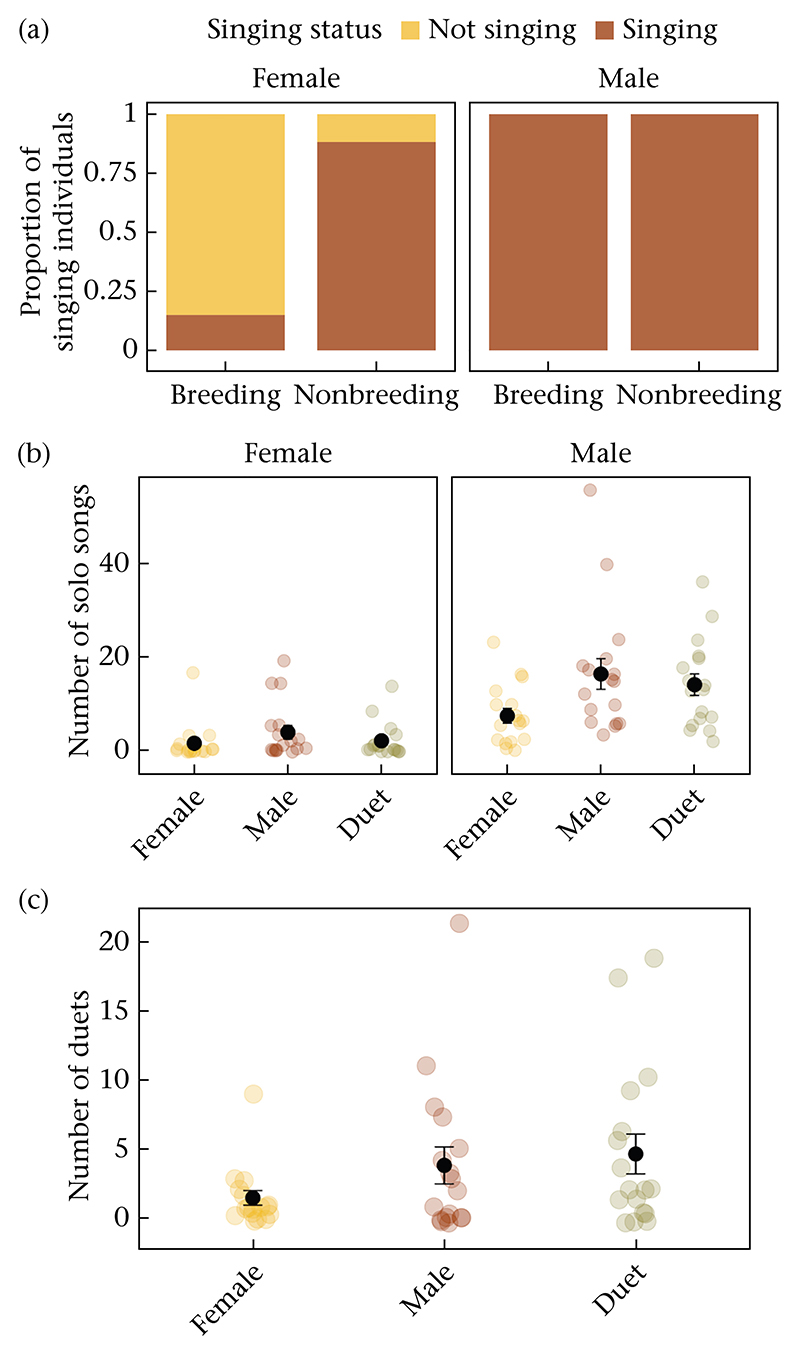
Songs produced in response to playbacks. (a) Proportion of female and male individuals that sang in response to at least one of the treatments. (b) Number of solo songs produced in response to playback treatments by each sex in the nonbreeding season. (c) Number of duets produced in response to playback treatments by each pair in the nonbreeding season. In panels b and c, coloured dots show each individual or pair, and black dots show the group mean and SE. PC1, principal component 1.

All males (*N* = 20 for the breeding season and *N* = 17 for the nonbreeding season) sang at least one song in response to play-backs during both seasons (Fig. 3a). In contrast, the number of singing females depended on the season (Chi-square test: χ*2*_1_ = 16.91, *P* < 0.001). Only 15% (3 of 20) of females responded with song during the breeding season, while 88% (15 of 17) responded with song during the nonbreeding season.

During the nonbreeding season, female birds sang most of their songs as duets (proportion of duets to all songs, *X* = 0.66, SD = 0.36), whereas males mostly sang solo songs (proportion of duets to all songs, *X* = 0.20, SD = 0.24). Of the 169 duets we recorded across trials, 143 (85%) were initiated by males. The number of solo songs produced in response to playback depended on sex, whereby males produced more solo songs than females (χ*2* = 24.97, *P* < 0.001, Table 2 and Fig. 3b). On average, females sang 2.4 solo songs per trial (SD = 4.7, range 0—19), whereas males sang 12.5 solo songs per trial (SD = 10.8, range 0—56). Females produced more solo songs in response to male intruders than duetting (contrast estimate = 0.65, *P* = 0.006) or female intruders (contrast estimate = 0.96, *P* = 0.0001). Males, however, sang an equal number of solo songs in response to male and duetting intruders (*P* = 0.2), which were both more than their response to female intruders (contrast estimates = 0.80 and 0.65, respectively, for male versus female and duet versus female comparisons, both *P* < 0.001, Table S5). Although the interaction effect between treatment and sex was statistically not significant (χ*2* = 4.66, *P* = 0.097). On average, pairs sang 3.3 duets per trial (SD = 4.95, range 0—21). For the number of duets, there was an order effect (χ*2* = 7, *P* = 0.03); pairs produced more duets in their third trial than in the second one (contrast estimate = 0.56, *P* = 0.02; all other comparisons not significant). Controlling for the influence of trial order, there was a significant effect of treatment (χ*2* = 19.34, *P* < 0.001, Table 3 and Fig. 3c). Pairs formed more duets in response to male and duet playbacks than female playback (male versus female contrast estimate = 0.84, *P* = 0.001; duet versus female contrast estimate = 1.01, *P* < 0.001, Table S6). Duet output of the resident pair to male and duet play-backs were similar (contrast estimate = 0.16, *P* = 0.57).

#### Association between aggression and song in males and females

**Figure 4:**
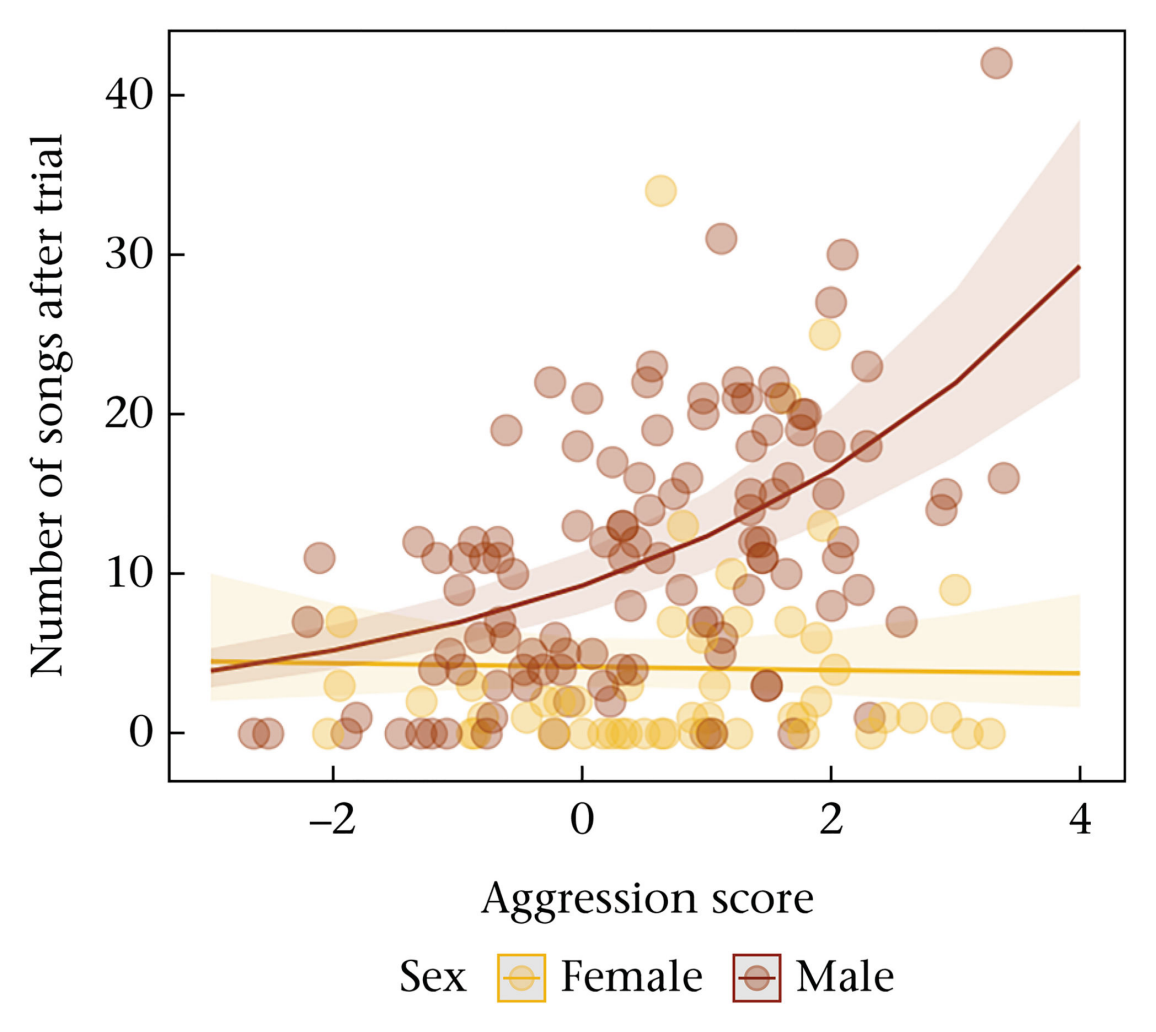
Relationship between the aggression score principal component 1 (PC1) and song output in the first 5 min after the playback for each sex. Males are represented by red and females are represented by yellow. Each coloured circle represents a single trial.

The association between aggressive behaviour during the trials and songs produced after the trial depended on sex (χ*2* = 7.71, *P* = 0.006). In males, song output was positively associated with aggression, whereas in females, there was no such relationship (Fig. 4).

### Female Song, Aggression, and Territory Retention

Of the 26 colour-banded females observed across years, seven lost their territory (in two of the seven, the female was missing while the male was still present, and in five of the seven, the pair was missing). For the nine colour-banded females that were tested in the playback experiment, neither the relationship between average song rate nor the probability of territory retention (χ*2* = 0.34, *P* = 0.56) nor the relationship between average aggression score and the probability of territory retention was significant (χ*2* = 1.45, *P* = 0.23). Nevertheless, it should be noted that the sample size for these analyses was quite small.

## Discussion

In this study, we tested the functions of female song in the Galápagos yellow warbler with a multiseasonal playback experiment. Our results showed a strong seasonal pattern in female singing and aggression in response to playbacks. Females responded to playbacks by singing and being aggressive, mostly in the nonbreeding season. In contrast to the North American sister species (Hobson & Sealy, 1990), Galápagos yellow warbler females mainly sang duet songs replying to their male partner. Moreover, females sang fewer solo or duet songs towards female song play-back than male song or duet playback, providing no support for the intrasexual competition hypothesis for singing. The absence of association between female aggressive behaviour and song output in response to playbacks suggests that female song (either solo or duet) does not signal threat in territory defence. Overall, territory loss occurred in seven of 26 females (27%), which is comparable to the proportion observed in females for which we have systematic playback data, where three out of nine (33%) lost their territories across years. Territory retention in the latter nine females was not explained by song rate or aggression scores in response to play-backs. We acknowledge that the small sample size limits the strength of these conclusions; therefore, our results should be interpreted with caution. Nevertheless, these findings provide no support for the territorial defence function of female song. In the following paragraphs, we discuss these results in the context of the species’ natural history and other possible functions of female song.

Although both sexes stay together in their territory year-round, and in some cases for multiple years, only the male yellow warblers sang and defended in response to playbacks in both seasons. Females, however, mainly sang and defended in the nonbreeding season. This discrepancy between sexes might be due to differential costs of singing. The same pattern was observed in another sedentary species of the family *Parulidae*, the rufous-capped warbler, *Basileuterus rufifrons*, in which females sang less often and were less aggressive in response to intruders during the breeding season, whereas males showed strong responses in both seasons (Demko & Mennill, 2018). In both species, females are the primary contributors to nest building and incubation, which may pose opportunity costs to singing and participating in territory defence. Alternatively, females might be keeping quiet due to higher relative nest predation risks of singing during breeding. For example, Kleindorfer et al. (2016) found that in superb fairy-wrens, *Malurus cyaneus*, female song rate predicted offspring predation rates, likely because females were singing closer to (and sometimes inside) the nest than males. Nevertheless, it is important to note that our data set represents only one breeding and one nonbreeding season. Additional replications with larger sample sizes will be required to draw stronger conclusions about seasonal consistency in these behavioural patterns.

Hobson and Sealy (1990) suggested that female song in the North American yellow warbler mainly functions in intrasexual competition for resources because they observed female song prior to nest building; two of four females that they recorded sang in response to a female decoy in their territory. Under this hypothesis, we predicted that females would be more aggressive and sing more towards female intruders. However, our results did not support this. During the nonbreeding season (when the female song was mainly present), females were equally aggressive towards all intruders, but they sang the least amount of duets and solo songs towards female intruders. This difference is paralleled in the singing patterns of the two sister species; females only were observed to sing solo songs in North America but females sang mostly duets in Galápagos. In a meta-analysis, Logue (2005) found that same-sex aggression bias was more common in nonduetting species, while duetting ones were more cooperative in defence, showing less bias towards the same sex. Nevertheless, experimental support for the intrasexual competition hypothesis in the North American species is also lacking.

Although aggression predicted song output in males, female aggression did not predict song output in response to intruder playbacks. This finding provides no support for the territory defence function of female song. Although territory defence may be the most common function of female song and duetting in the systems studied so far (Barros et al., 2024; Hall, 2004), this is not the case for all species. In the eastern bluebird, *Sialia sialis*, females did not increase song rates in response to intruders, even though they were aggressive towards intruders; instead, female songs were mainly used as an intrapair communication signal during intrusions (Rose et al., 2019). Similarly, in the bay wren, *Thryothorus nigricapillus*, removal of a single pair member did not lead to loss of territory, indicating that duets are not essential for maintaining territorial defence (Levin, 1996). Nevertheless, our study design does not allow us to rule out the possibility that the female song might be used in the presence of intruders as a pair-directed signal to coordinate defence. Female yellow warblers could be singing to signal their location to their pair during an intrusion, either to increase proximity or prevent misdirected aggression (Dahlin & Benedict, 2014). Additionally, female solo songs and duets may still contribute to territory defence indirectly by signalling territory ownership and reducing the likelihood of intrusion and usurpation attempts.

We predicted that duetting pair intruders would receive the strongest aggressive response from both sexes; however, this was not the case. Females were equally aggressive towards all intruders, and males were equally and more aggressive towards males and duetting intruders than the female intruders. It is possible that the use of a single speaker for a duetting pair was perceived as unnatural (Koloff & Mennill, 2013). Nonetheless, given that we often observed pairs duetting as close as <1 m to each other in our study population, we do not believe the experimental set-up was unrealistic. Instead, we speculate that if a solo male intruder already poses a very high threat, the pair might already be showing aggression at their maximum capacity, resulting in a ceiling effect.

Given the lack of support for either the intrasexual competition or the territory defence hypothesis, female song and duetting in Galápagos yellow warblers could be a mate-directed signal used to coordinate the social activity of the pair or maintain the pair bond. More specifically, pairs may form duets to maintain contact with each other or signal commitment to the partnership (Hall, 2004). In the former case, we would expect pairs to be visually separated when duetting. In the latter, we would expect, for example, duetting to be negatively linked with extra-pair mating. Additionally, as noted in the previous paragraphs, duets may facilitate cooperation during territory defence. With this hypothesis, we expect pair proximity during defence to correlate with duet output. We are currently testing these hypotheses experimentally as well as with observational data.

In summary, we show that female song is very common in the year-round resident Galápagos yellow warblers. To our knowledge, this is the first documentation of female song in this species after the recent *petechia*—*aestiva* taxonomic split, despite Galápagos being a long-standing hotspot for ornithological research. Our findings reveal the importance of studying birdsong in multiple seasons, evaluating the functions of both female and male song, and considering multiple hypotheses when interpreting the evolution of vocal communication.

## Supplementary Material

Supplementary material associated with this article is available at https://doi.org/10.1016/j.anbehav.2026.123483.

Suplementary

## Figures and Tables

**Figure 1:**
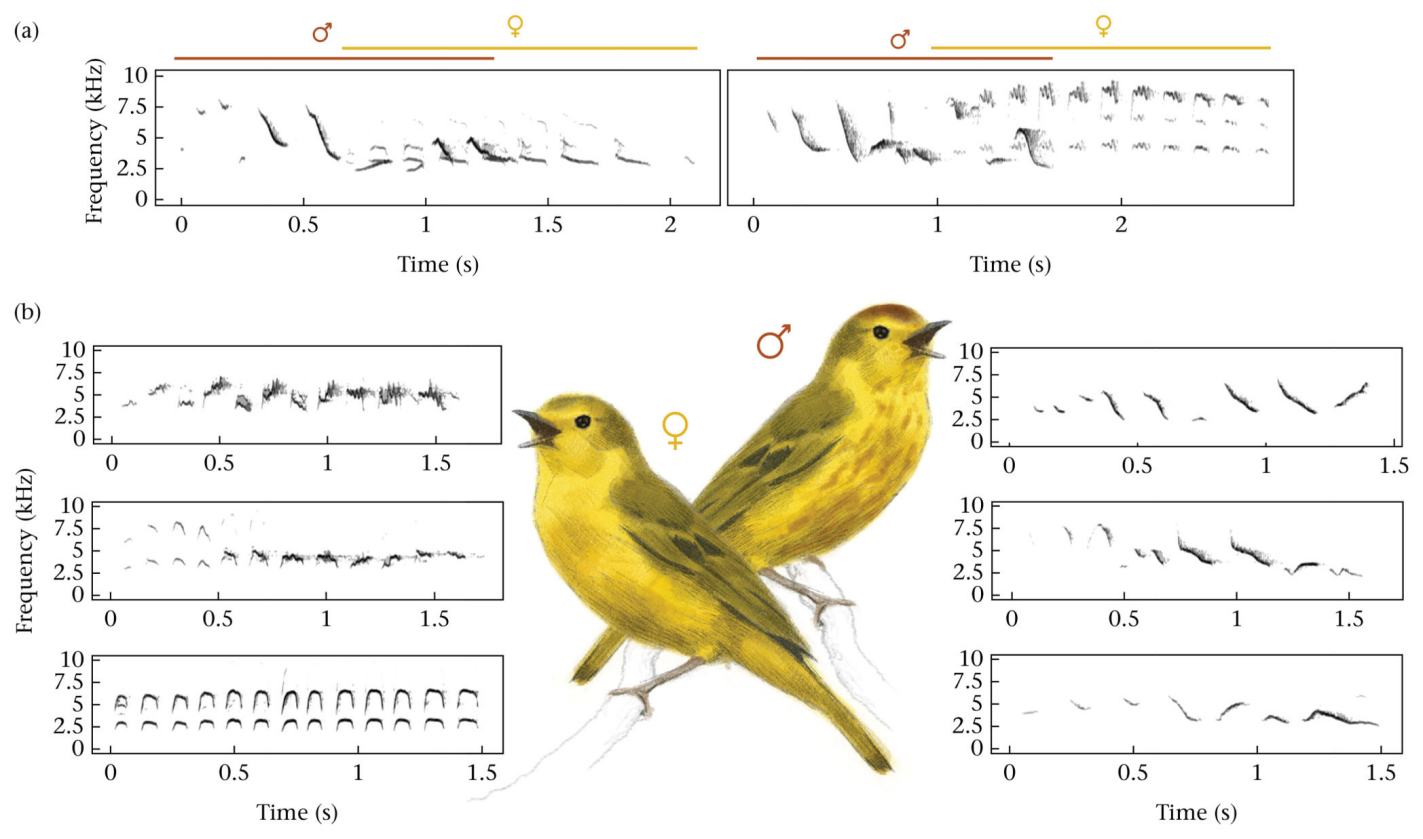
Illustration of female and male yellow warblers and their respective songs. (a) Spectrograms of two duets are shown with male parts marked in red and female parts marked in yellow. (b) Three female (left) and three male (right) songs. All displayed songs are obtained from different individuals. Spectrograms are created using 512 samples per window and 99% overlap with the package seewave in R (Sueur et al., 2008). Illustrations are courtesy of Lena Gies.

**Table 1:** Output of linear mixed model for the aggression score

Predictors	Estimate	95% CI	χ*^2^*	*df*	*P*
**(Intercept)**	**–1.58**	**–2.12 to –1.04**	**33.785**	**1**	**<0.001**
Treatment (male)	**–**0.01	–0.61 to 0.60	0.589	2	0.745
Treatment (duet)	0.20	–0.41 to 0.81			
**Sex (male)**	**0.81**	**0.08**–**1.53**	**4.852**	**1**	**0.028**
**Season (nonbreeding)**	**2.47**	**1.77**–**3.16**	**49.084**	**1**	**<0.001**
**Treatment (male) × sex (male)**	**1.42**	**0.55**–**2.28**	**11.186**	**2**	**0.004**
**Treatment (duet) × sex (male)**	**1.02**	**0.16**–**1.88**			
Treatment (male) × season (nonbreeding)	**–**0.67	–1.57 to 0.23	2.460	2	0.292
Treatment (duet) × season	**–**0.11	–1.01 to 0.79			
(Nonbreeding)					
**Sex (male) × season (nonbreeding)**	**–1.52**	–**2.49 to** –**0.55**	**9.533**	**1**	**0.002**
(Treatment (male) × sex (male)) × season (nonbreeding)	0.69	– 0.58 to 1.96	1.567	2	0.457
(Treatment (duet) × sex (male)) × season (nonbreeding)	**–**0.02	–1.29 to 1.25			

Bold values indicate statistical significance of the overall term at α set to 0.05.

**Table 2:** Output of the generalized linear mixed model for the number of solo songs

Predictors	Estimate	95% CI	χ*^2^*	*df*	*P*
(Intercept)	–0.71	-1.45 to 0.03	3.50	1	0.062
**Treatment (male)**	**0.96**	**0.49**–**1.42**	**19.87**	**2**	**<0.001**
**Treatment (duet)**	**0.31**	**-0.21 to 0.83**			
**Sex (male)**	**2.50**	**1.56**–**3.44**	**26.95**	**1**	**<0.001**
Treatment (male) × sex (male)	–0.16	–0.66 to 0.35	4.66	2	0.097
Treatment (duet) × sex (male)	–0.34	–0.22 to 0.90			

Bold values indicate statistical significance of the overall term at α set to 0.05.

**Table 3:** Output of the generalized linear mixed model for the number of duets

Predictors	Estimate	95% CI	χ*^2^*	*df*	*P*
(Intercept)	0.07	–0.60 to 0.73	0.041	1	0.839
**Treatment (male)**	**0.84**	**0.38**–**1.30**	**19.342**	**2**	**<0.001**
**Treatment (duet)**	**1.01**	**0.56**–**1.46**			
**Treatment order (second)**	–**0.36**	–**0.80 to 0.07**	**7.00**	**2**	**0.03**
**Treatment order (third)**	**0.20**	–**0.15 to 0.54**			

Bold values indicate statistical significance of the overall term at α set to 0.05.

## Data Availability

Data and R script to reproduce analyses can be found in the Supplementary material.
