# Supplementary material for: Solo songs, duets and territory defence across seasons in female Galápagos yellow warblers, Setophaga petechia aureola
Source: Anim Behav. Author manuscript; Available in PMC 2026 May 20. (PMC7619094; doi:10.1016/j.anbehav.2026.123483)
Supplement: Suplementary [file EMS213482-supplement-Suplementary.zip › 1-s2.0-S0003347226000205-mmc1.docx]

**Supplementary material**

**Table S1**

Colour band status of pairs in both seasons

| Status | Breeding season | Nonbreeding season |
| --- | --- | --- |
| Only M banded | 1 | 1 |
| Only F banded | 1 | 2 |
| Both pair members banded | 6 | 8 |
| No pair members banded | 12 | 6 |
| Total | 20 | 17 |

F: female; M: male

**Table S2**

Number of pairs that received treatments in the corresponding order in each season

| Treatment order | Nonbreeding season | Breeding season |
| --- | --- | --- |
| M-F-duet | 3 | 3 |
| M-duet-F | 3 | 3 |
| F-M-duet | 3 | 4 |
| F-duet-M | 3 | 3 |
| Duet-M-F | 3 | 3 |
| Duet-F-M | 2 | 3 |

F: female; M: male

**Table S3**

Factor loadings to principal component 1 (Aggression Score) for each variable

| Response variable | Factor loading |
| --- | --- |
| Flights | −0.51 |
| Time spent within 5 m of the speaker | −0.50 |
| Closest approach to the speaker | 0.53 |
| Latency to respond to playback | 0.44 |

**Table S4**

Post hoc pairwise comparisons of the aggression score linear mixed model

| Contrast | Season | Estimate | SE | *df* | *t* | *P* |
| --- | --- | --- | --- | --- | --- | --- |
| F F - M F | Breeding | 0.005 | 0.309 | 154.677 | 0.017 | 1 |
| F F - duet F | Breeding | −0.203 | 0.309 | 154.677 | −0.656 | 0.986 |
| F F - F M | Breeding | −0.809 | 0.368 | 115.864 | −2.197 | 0.247 |
| **F F - M M** | **Breeding** | **−2.22** | **0.368** | **115.864** | **−6.026** | **0** |
| **F F - duet M** | **Breeding** | **−2.031** | **0.368** | **115.864** | **−5.512** | **0** |
| M F - duet F | Breeding | −0.208 | 0.309 | 154.677 | −0.673 | 0.985 |
| M F - F M | Breeding | −0.814 | 0.368 | 115.864 | −2.21 | 0.241 |
| **M F - M M** | **Breeding** | **−2.225** | **0.368** | **115.864** | **−6.04** | **0** |
| **M F - duet M** | **Breeding** | **−2.036** | **0.368** | **115.864** | **−5.526** | **0** |
| Duet F - F M | Breeding | −0.606 | 0.368 | 115.864 | −1.646 | 0.57 |
| **Duet F - M M** | **Breeding** | **−2.017** | **0.368** | **115.864** | **−5.476** | **0** |
| **Duet F - duet M** | **Breeding** | **−1.828** | **0.368** | **115.864** | **−4.961** | **0** |
| **F M - M M** | **Breeding** | **−1.411** | **0.309** | **154.677** | **−4.569** | **0** |
| **F M - duet M** | **Breeding** | **−1.221** | **0.309** | **154.677** | **−3.955** | **0.002** |
| M M - duet M | Breeding | 0.19 | 0.309 | 154.677 | 0.614 | 0.99 |
| F F - M F | Nonbreeding | 0.672 | 0.335 | 154.677 | 2.006 | 0.344 |
| F F - duet F | Nonbreeding | −0.092 | 0.335 | 154.677 | −0.273 | 1 |
| F F - F M | Nonbreeding | 0.711 | 0.397 | 126.003 | 1.792 | 0.475 |
| F F - M M | Nonbreeding | −0.719 | 0.397 | 126.003 | −1.813 | 0.461 |
| F F - duet M | Nonbreeding | −0.375 | 0.397 | 126.003 | −0.946 | 0.934 |
| M F - duet F | Nonbreeding | −0.763 | 0.335 | 154.677 | −2.279 | 0.209 |
| M F – F M | Nonbreeding | 0.039 | 0.397 | 126.003 | 0.098 | 1 |
| **M F - M M** | **Nonbreeding** | **−1.391** | **0.397** | **126.003** | **−3.507** | **0.008** |
| M F - duet M | Nonbreeding | −1.047 | 0.397 | 126.003 | −2.639 | 0.095 |
| Duet F - F M | Nonbreeding | 0.802 | 0.397 | 126.003 | 2.022 | 0.336 |
| Duet F - M M | Nonbreeding | −0.628 | 0.397 | 126.003 | −1.583 | 0.612 |
| Duet F - duet M | Nonbreeding | −0.284 | 0.397 | 126.003 | −0.715 | 0.98 |
| **F M - M M** | **Nonbreeding** | **−1.43** | **0.335** | **154.677** | **−4.27** | **0** |
| **F M - duet M** | **Nonbreeding** | **−1.086** | **0.335** | **154.677** | **−3.242** | **0.018** |
| M M - duet M | Nonbreeding | 0.344 | 0.335 | 154.677 | 1.028 | 0.908 |

Bold values indicate statistical significance at α set to 0.05. F: female; M: male

**Table S5**

Post hoc pairwise comparisons of the solo song model

| Contrast | Sex | Estimate | SE | *Z* ratio | *P* |
| --- | --- | --- | --- | --- | --- |
| **F - M** | **F** | **−0.956** | **0.235** | **−4.060** | **0.0001** |
| F - duet | F | −0.307 | 0.263 | −1.167 | 0.473 |
| **M - duet** | **F** | **0.648** | **0.212** | **3.062** | **0.006** |
| **F - M** | **M** | **−0.799** | **0.108** | **−7.422** | **<0.0001** |
| **F - duet** | **M** | **−0.648** | **0.110** | **−5.872** | **<0.0001** |
| M - duet | M | 0.151 | 0.088 | 1.714 | 0.200 |

Bold values indicate statistical significance at α set to 0.05.

F: female; M: male

**Table S6**

Post hoc pairwise comparisons of the duet model

| Contrast | Estimate | SE | *Z* ratio | *P* |
| --- | --- | --- | --- | --- |
| **F - M** | **−0.841** | **0.236** | **−3.568** | **0.001** |
| **F - duet** | **−1.010** | **0.231** | **−4.372** | **<0.001** |
| M - duet | −0.170 | 0.168 | −1.012 | 0.569 |

Bold values indicate statistical significance at α set to 0.05. F: female; M: male


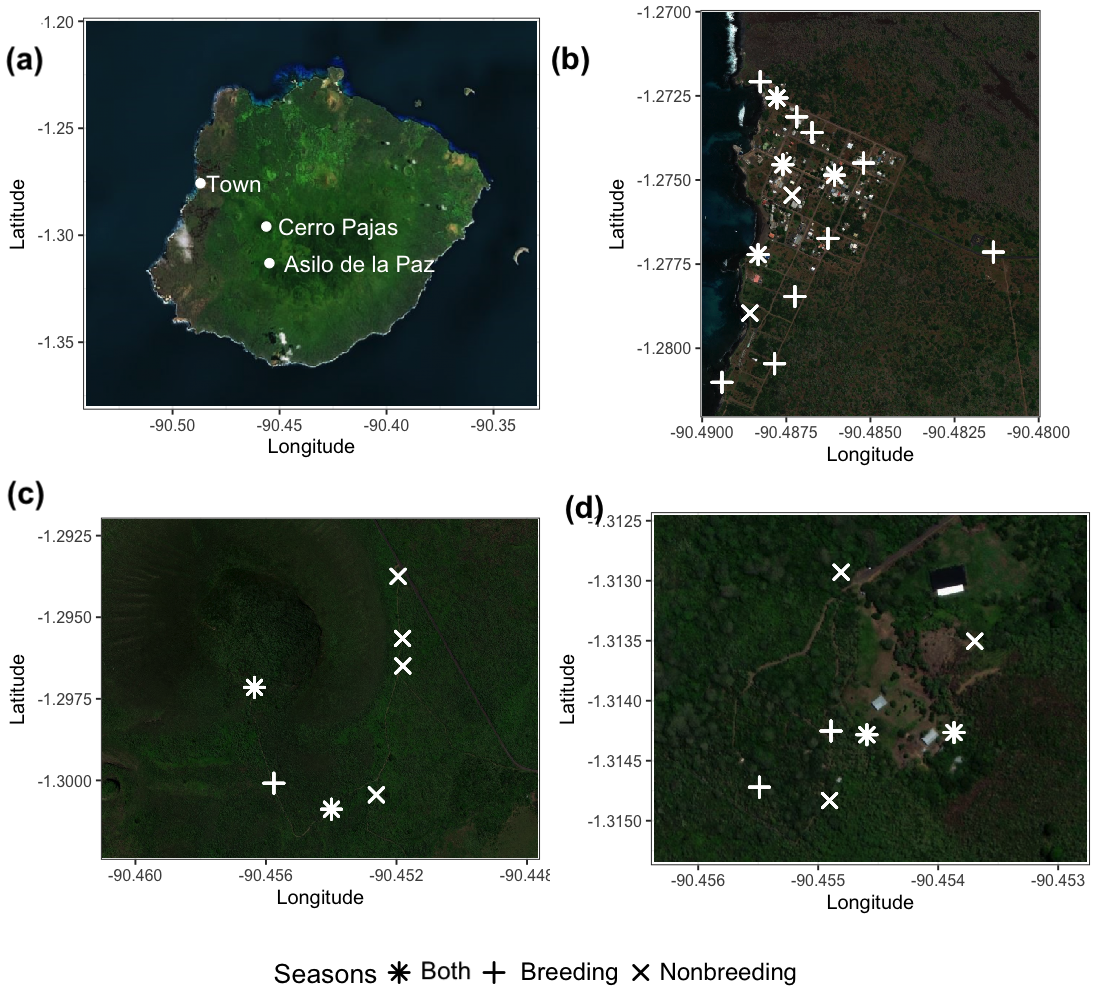


**Figure S1.** Satellite maps of study sites and locations of the playback experiments. (a) Three study sites are shown on Floreana Island, (b) Town, (c) Cerro Pajas, and (d) Asilo de la Paz. Each study territory is shown on maps b, c, and d with white symbols; their shape indicates the season in which the experiments were performed.
